# Genotype for hypocretin receptor (*hcrtr2*) affects appetite in zebrafish^☆^

**DOI:** 10.1016/j.ygcen.2025.114808

**Published:** 2025-09-02

**Authors:** Nathan Lewandowski, Miranda Brainard, Chelsea Kalb, Ashley Wong, Qin Liu, Richard Londraville

**Affiliations:** University of Akron, Department of Biology Akron, OH 44325-3908, United States

**Keywords:** Feeding assay, Appetite, Zebrafish, Hypocretin, Genotyping

## Abstract

We investigated the role of hypocretin receptor in signaling appetite in zebrafish (*Danio rerio*). Hypocretin is a small neuropeptide known for its effects on circadian rhythm and appetite. Wild-type and heterozygous hu2098 (knockout for *hcrtr2*) zebrafish were raised to adulthood (3–4 months post fertilization) and genotyped. Feeding rate was measured directly using a novel technique that analyzed images of fish feeding continuously on brine shrimp, in which all individual brine shrimp were identified in a tank with a feeding fish. Fish were food restricted for 19–29 h before a feeding session, and feeding rate was determined by the regression of brine shrimp consumed/min over an eight-minute feeding period. Utilizing a mixed-effects ANCOVA model and accounting for mass as a covariate, heterozygous fish (*hcrtr*2^+^_/_*hcrtr*2^−^) ate brine shrimp at a significantly faster rate (mean 23.4 ± 12.6 shrimp/min, n = 12) than wildtype fish (*hcrtr*2^+^_/_*hcrtr*2^+^) (20.5 ± 13.8 shrimp/min, p = 0.033, n = 11) These results support a role for *hcrtr2* in appetite regulation.

## 1. Introduction

Hypocretin, also known as orexin, is a small neuropeptide that plays a multifunctional role in both central and peripheral nervous systems. Two orexigenic neuropeptides were first identified more than two decades ago in rats where expression was localized in the lateral hypothalamus and surrounding areas (de Lecea et al., 1998). Two isoforms of hypocretin (*hcrt*) were identified via the discovery of two G-Protein Coupled Receptors (GPCRs) that had unknown ligands (Sakurai et al., 1998). The first was named Hcrt1 with a length of 33 amino acids and the second as Hcrt2 at 28 amino acids (Yokobori et al., 2011). Both neuropeptides have corresponding GPCRs named Hcrtr1 and Hcrtr2 with varying affinities for each receptor (Yokobori et al., 2011). The neuropeptides were initially given the name orexin due to their stimulating feeding pathways (Sakurai et al., 1998).

Many feeding pathways interact in the vertebrate central nervous system. Neurons in the lateral hypothalamus (LH) that release Hcrt innervate neuropeptide Y (NPY) neurons; Hcrt release also upregulates NPY receptor availability (van den Pol et al., 1998). When NPY is blocked, feeding and appetite are suppressed even while hcrt is released from the LH, showing that NPY is necessary for normal feeding behavior (Jain et al., 2000). Leptin plays an anorexigenic role opposite to that of Hcrt and NPY by decreasing appetite and increasing fat metabolism (Gruzdeva et al., 2019). Hcrt neurons are reversibly inhibited by leptin through gamma-aminobutyric acid (GABA), therefore reducing the appetite stimulating effects of Hcrt (Burt et al., 2011). Secondly, leptin can inhibit Hcrt signaling through neurons releasing the neuropeptide neurotensin (NTS) that primarily express leptin receptor B (LepRb) within the lateral hypothalamus (Brown et al., 2017). In zebrafish, when NTS neurons expressing LepRb are activated, they inhibit Hcrt by suppressing Hcrt mRNA transcription (Levitas-Djerbi et al., 2015).

Hcrt is expressed ubiquitously among vertebrates (mice, goldfish, chickens, and zebrafish; Yokobori et al., 2011). Most of the research focus for Hcrt is on sleeping disorders such as insomnia and narcolepsy (Prober et al., 2006). Knockout of the *Hcrt* gene in mice and rats decreases overall neuronal activity and induces excessive drowsiness during the day, which are both symptoms of narcolepsy (Siegel, 2004). This result promoted the hypothesis that Hcrt plays a role in regulating the sleep-wake cycle and wakefulness in general. This was supported by increased Hcrt expression causing disruption in sleep as well as increased anxiety and excessive motor movement in zebrafish (Prober et al., 2006). Hcrt is now considered a modulator for wakefulness in zebrafish (Levitas-Djerbi and Appelbaum, 2017), but its role in appetite is largely unexplored, and the physiology of the hcrt receptor (Hcrtr2) is understudied. Unlike the hypocretin ligand, its receptor is expressed as a single allele (hcrtr2; Dyachuk, 2024). In this study we measured zebrafish appetite using a novel assay that measures consumption rate, rather than simply an endpoint of total consumption. We demonstrate that heterozygous knockout zebrafish for Hcrtr2 (*hcrtr*2^+^_/_*hcrtr*2^−^) consume brine shrimp at a significantly higher rate than WT fish, supporting a role for Hcrt signaling in zebrafish appetite signaling.

## 2. METHODS

### 2.1. Fish Husbandry

Fish were maintained at 28.0 °C, 14L:10D and fed brine shrimp or Ziegler zebrafish mash daily in the University of Akron Research Vivarium (UARV). Hypocretin receptor 2 (*hcrtr2*) heterozygous embryos (*hcrtr*2^+^_/_*hcrtr*2^−^) *f*_1_ were received from the Zebrafish International Resource Center (ZIRC) and were raised in the UARV zebrafish nursery. While being grown to adulthood, fish were stocked at 5–30 fish in each tank. For genotyping, fish were separated into individual tanks. Afterwards, fish were grouped in tanks by genotype and stocked at 2–6 fish per tank. Experiments were approved by The University of Akron IACUC.

### 2.2. Genotyping

**Fig. 1.**
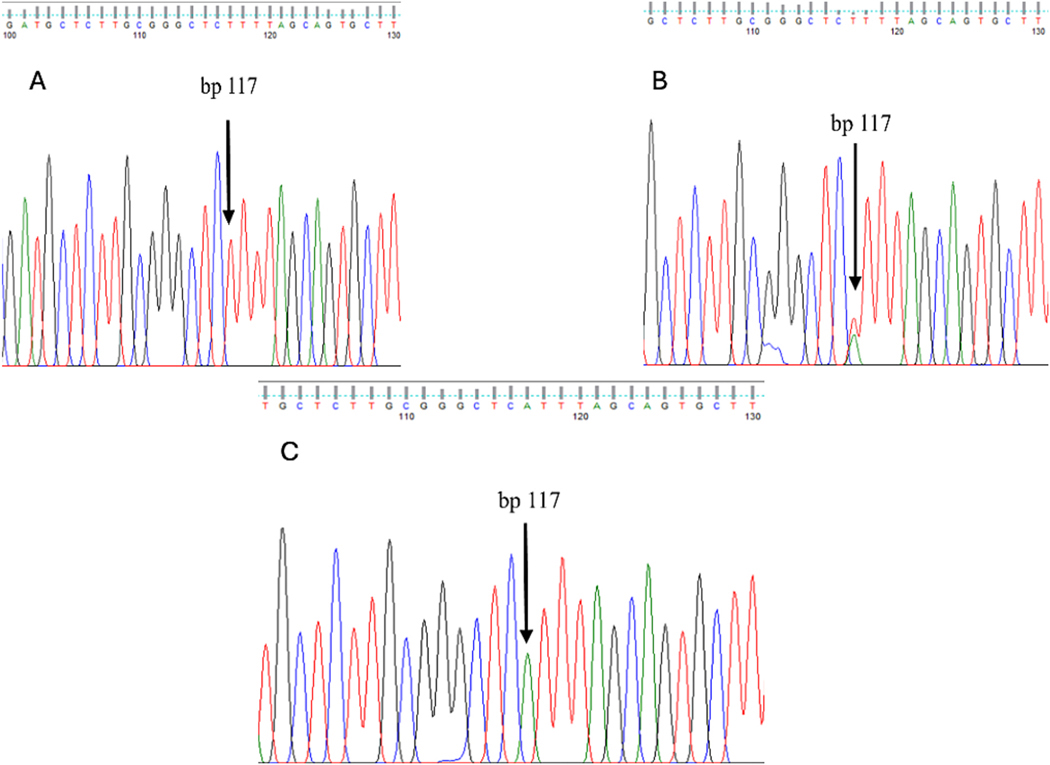
(**A)** Wild-type genotype zebrafish sequence (*hcrtr*2^+^/*hcrtr*2^+^). Thymine signal detected at base pair 117. **(B)** Heterozygous genotype zebrafish sequence (*hcrtr*2^+^/*hcrtr*2^−^). Thymine and adenine signal detected at base pair 117. **(C)** Homozygous mutant genotype. Adenine signal detected at base pair 117. Genotyped zebrafish sequence (*hcrtr*2^−^/*hcrtr*2^−^).

Zebrafish were transferred to individual 15 ml conical tubes filled with system water from their same tank. Water was then reduced to 5 ml and 5 μl of proteinase K (1 μg/ml) was added to each tube (Zhang, 2020). After a 20-minute incubation, fish were returned to their individual tanks and DNA was extracted from the incubation water using the Quick-DNA Miniprep Plus Kit (D4068 Zymo Research, Irvine, California). PCR was performed with the isolated genomic DNA with primers flanking the *hcrtr2* gene mutation (**F** 5′ GCCACTGCTCATCACAGACG 3′, **R** 5′ TGTGTACGTTTCTATCTCTCCTGTT 3′, 278 BP) and evaluated with 3 % agarose gel. Fluorescent gel bands were excised from the gels and the PCR product purified using the Zymoclean Gel DNA Recovery Kit (D4002 Zymo Research, Irvine, California). PCR products were sequenced at the University of Illinois Core Sequencing Facility to determine genotype of individual fish (Zhang et al., 2020) (Fig. 1).

### 2.3. Brine shrimp Preparation

Brine shrimp (*Artemia salina*) were prepared by adding 1 teaspoon of freeze-dried cysts to a saltwater solution and aerated for 36–48 h. Fresh brine shrimp were harvested in a fine mesh net and transferred to a 400 ml plastic beaker containing 350 ml tank water and a magnetic stir bar. The beaker was placed on a stir plate and brine shrimp were suspended slowly in the beaker. Individual aliquots of brine shrimp solution (1 ml) were made daily.

### 2.4. Zebrafish feeding apparatus

**Fig. 2.**
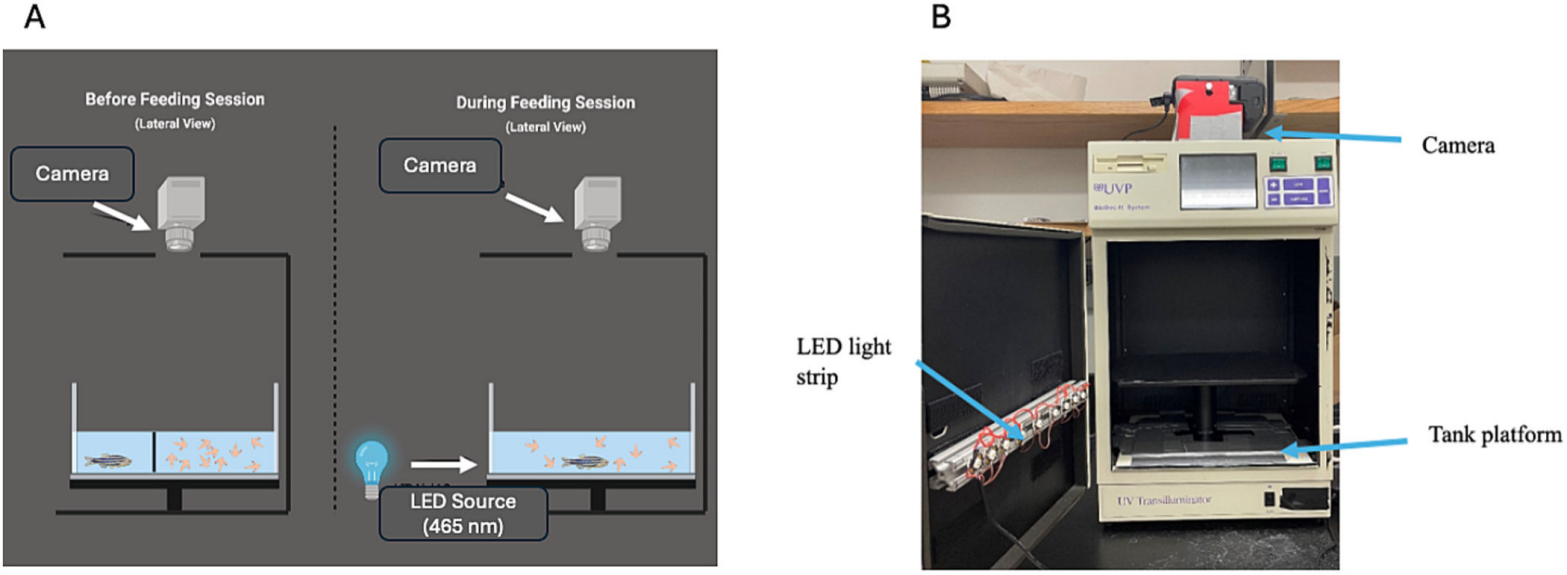
Feeding apparatus schematic and photo of actual device. The door (on left with LED strip) is closed during the assay, and the LEDs provide all illumination.

A gel imaging system was modified to control light exposure and for image acquisition during feeding. Nine blue 465 nm LEDs were glued to a 27 cm aluminum rod and soldered together in a daisy chain and powered by a 3V 1 amp power adapter. The emission of these LEDs (465 nm) is well within the visual spectrum for zebrafish (340–640 nm; Risner et al. 2006). The rod was attached to the apparatus door with hook and loop fasteners at the same level of the water in the feeding tank to illuminate the water evenly. A 10-ohm resistor was soldered in-between the power source and the daisy chain of LEDs on the aluminum rod to decrease the brightness of the blue light. A Canon EOS 6D camera (Canon USA, Melville, NY) was mounted on top of the apparatus and focused through a 5.5 cm diameter opening. The feeding tank was placed on an adjustable platform so the entire feeding tank could be put in focus and in frame. A JJC photo timer (TC-80 N3; JJC Shenzhen, China) was attached to automatically take photos of the feeding tank during experimentation (Fig. 2).

### 2.5. Feeding assay

The experimental group contained 12 heterozygous fish (*hcrtr*2^+^_/_*hcrtr*2^−^) and the control group contained 11 wild-type fish (*hcrtr*2^+^_/_*hcrtr*2^+^). One homozygous fish (*hcrtr*2^−^/*hcrtr*2^−^) was identified prior to experimentation but was not included in data analysis. Each fish was weighed on the first and last days of the experiment by filling a 400 ml plastic beaker with approximately 100 ml of tank water which was tared. Before each trial the feeding tank (26 cm × 18.5 cm × 8.5 cm) was filled with 500 ml of fresh tank water at 26–28°C. Brine shrimp were illuminated by the blue LED light shining on the fish tank during feeding (Fig. 2, Supplemental Fig. S1). A divider was placed in the tank and a zebrafish was placed on the left side of the divider. The tank was transferred to the feeding apparatus and the blue LED lights lit. Zebrafish were given three minutes to acclimate before the feeding trial started. An aliquot of brine shrimp solution was pipetted into the water on the center-right side of the divider. After a preliminary image was taken, the divider was removed, and the apparatus door was closed, immediately after which the photo timer began. The fish was allowed to feed over an eight-minute trial with an image being taken every minute (preliminary trials showed that feeding plateaued after 8 min). The tank was then removed from the apparatus and the fish was put back into its storage tank. This was repeated daily for all 24 fish over 14 days. Fish were food restricted for 19–29 h before a feeding session and the order of fish assayed was randomized each day.

### 2.6. Software

**Fig. 3.**
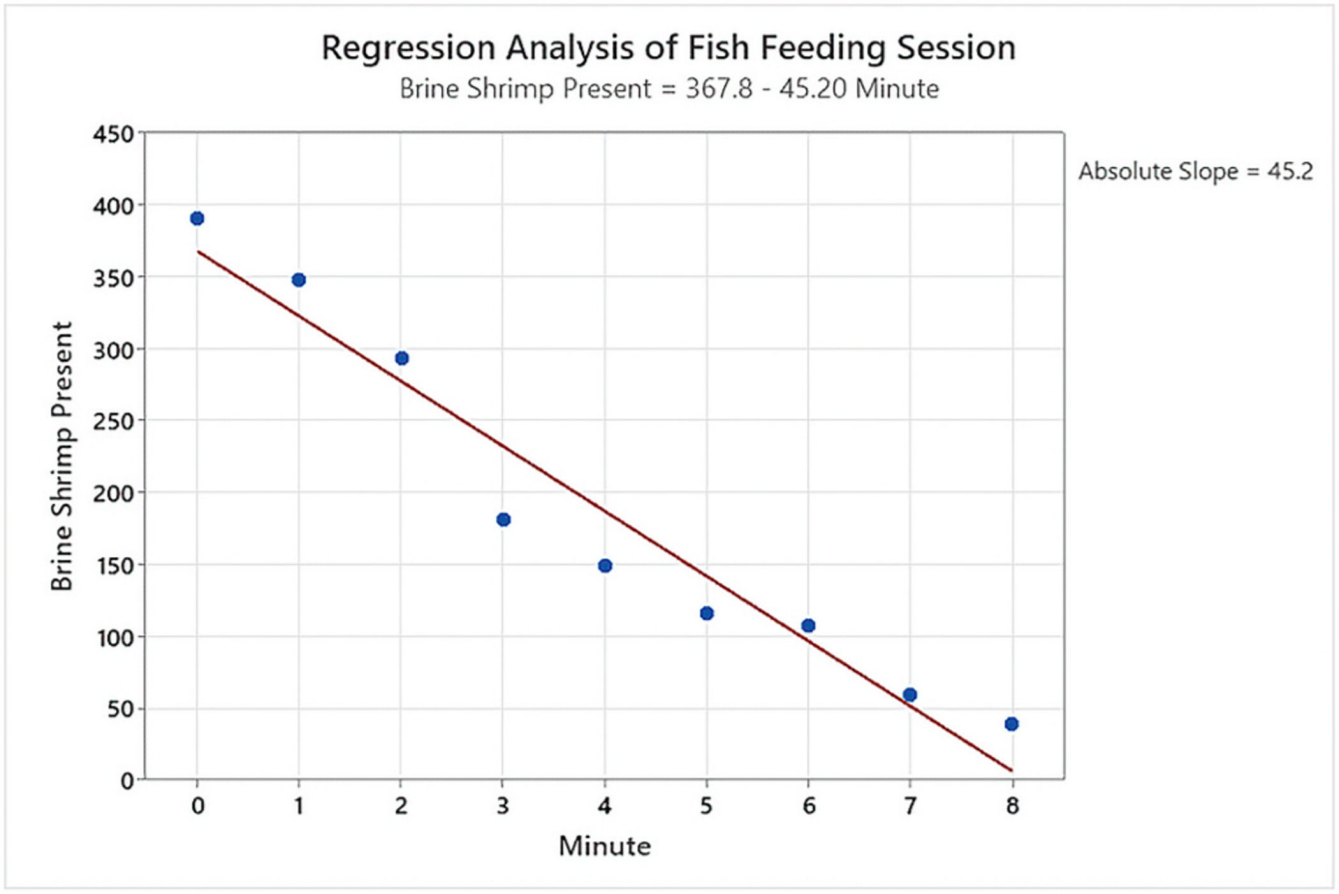
Example of one trial with a single fish. Total number of brine shrimp detected by the software decreases linearly over the duration of the assay.

Blue LEDs illumination made the brine shrimp brighter than any of the background pixels in the images. Python code was written (Lewandowski, 2024) to convert each picture into a grayscale image, ranking pixels on a scale of zero to one where zero is black and one is white. A threshold was set to count only the objects that were above the set threshold and the objects were counted and presented as output. This threshold was determined through initial trials and then standardized by running images through the program with known numbers of brine shrimp present in the tank. Trials were then plotted as brine shrimp detected by the software over time (Fig. 3). From this plot, we computed the slope which is *rate of consumption* (rather than simply amount consumed).

### 2.7. Statistics

Consumption rate (brine shrimp/min) was calculated by regression analysis of brine shrimp present throughout each feeding session from zero to eight minutes. Consumption rate between genotypes was analyzed comparing slopes using a mixed effects model ANCOVA with Minitab Statistical software (State College, PA). Fish mass was incorporated in the model as a covariate to account for differences in consumption based on size. Minute-by-minute feeding rate was analyzed using post-hoc Tukey’s analysis.

## 3. Results

### 3.1. Feeding assay

**Fig. 4.**
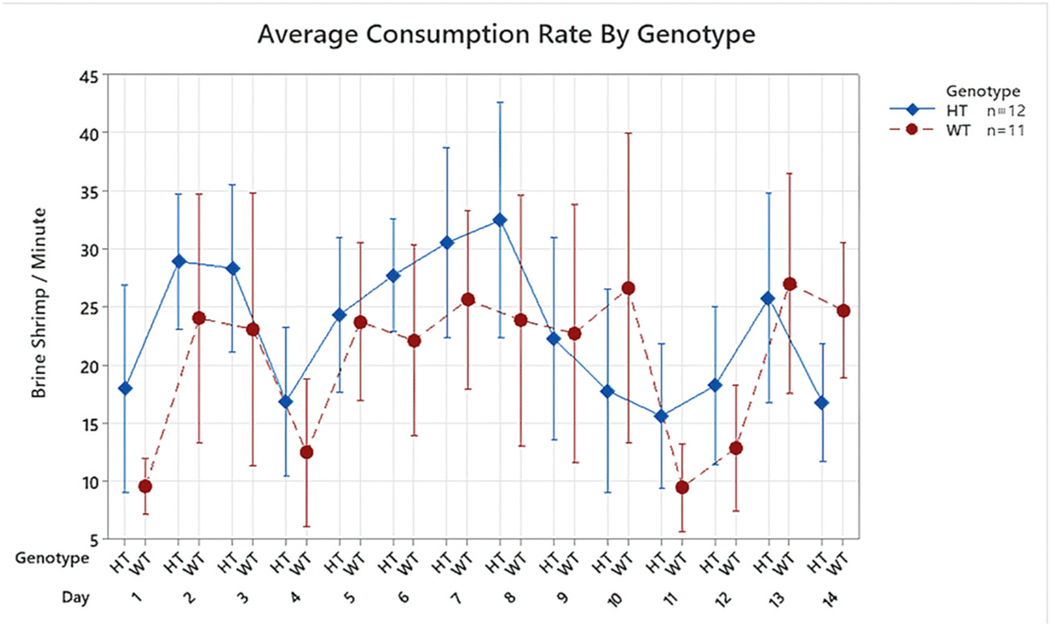
Days 1 through 14 average consumption rate by genotype. Heterozygous fish (HT) consume brine fish faster (mean 23.4 ± 12.6 shrimp/min, n = 12) than wildtype (WT) fish (20.5 ± 13.8 shrimp/min, n = 11, p = 0.033).

Brine shrimp consumed per minute decreased linearly over the time period of the assay (Fig. 3). Slopes were transformed (square root) and normality was assessed (Ryan-Joiner, p = 0.100). Homoscedasticity was confirmed with Levene’s test (p = 0.201, Supplemental Fig. S2) prior to ANCOVA (Fig. 4, Table 1). Day was a significant source of variation (p = 0.02) and was included as a random effect in the mixed effects ANCOVA. The overall consumption rate for heterozygous fish (n = 12) was significantly higher than wild-type (n = 11) p = 0.027 (Table 1).

Furthermore, heterozygous fish on average consumed more brine shrimp/minute on 10 of the 14 days compared to wild type (Fig. 4). Minute-by-minute consumption rate was highest in minutes 1 and 2 over the 8 min assay, but was not different by genotype (Table 2). Gender did not have a significant effect on consumption rate (8.48 ± 1.11 brine shrimp/min males, 17.52 ± 4.2 females p = 0.054) and the interaction between gender and treatment was not significant (p = 0.953, Supplementary Fig. S3). Zebrafish mass (113 ± 10.2 mg males and 125 ± 9.8 mg females), did not significantly impact consumption rate (p = 0.163; Table 1). For the one homozygous mutant, feeding rate appeared significantly higher compared to the other two genotypes. Grubbs test indicated that none of the homozygous mutant consumption rates were outliers (Fig. 4).

## 4. Discussion

Here we demonstrate that *hcrtr2* copy number affects appetite for the first time, using a novel assay. Heterozygous fish consumed brine shrimp at a higher rate (23.4 brine shrimp/min vs. 20.5 brine shrimp/min for WT) and more total brine shrimp during the trial (on average, heterozygotes consumed 24 more brine shrimp per trial than wild type). The one homozygous mutant identified had an intake of 35.8 brine shrimp/ min. The overall trend of an increase in appetite as *hcrtr2* copy number decreases counters previous evidence that intracerebroventricular injections of hypocretin stimulate appetite in zebrafish and other vertebrates (Yokobori et al., 2011, Panula 2010).

The neuronal network between leptin, NTS, and Hcrt may contribute to the phenotype we observed. Both NTS and the Hcrt-sensitive neurons are primarily colocalized within the hypothalamus with a majority of the Hcrt neurons expressing the receptor for NTS (Furutani et al., 2013). Leptin operates via NTS neurons expressing LepRb (leptin sensitive) to inhibit Hcrt neuron excitation and suppress *hcrt* transcription (Levitas-Djebi et al., 2015, Brown et al., 2017). Decreased expression of Hcrt neurons increases the expression of Hcrtr2, so the loss of Hcrt-sensitive neurons increases *hcrt* receptor transcription (Elbaz et al., 2012). Lower Hcrtr2 expression could result in higher expression of Hcrt. We cannot say if the *hcrtr*2^+^/*hcrtr*2^−^ genotype results in decreased Hcrtr2 expression (or decreased Hcrtr2 expression on the plasma membrane), but it is possible that decreased Hcrtr2 increases Hcrt expression within the lateral hypothalamus (Elbaz et al., 2012). Fewer NTS neurons expressing LepRb with increased Hcrt-sensitive neurons could have diminished the anorexigenic phenotype of leptin (Brown et al., 2017).

Here we describe a new method of quantifying adult fish feeding. Many methods have been generated for measuring fish appetite, including UV–vis spectrometry, fluorescently labeled food, and even manually counting pellets consumed by each fish (Mashhadi et al., 2020, Jordi et al., 2015, Yokobori et al., 2011). This assay measures brine shrimp intake throughout an entire feeding session, rather than just an endpoint assay. We documented that consumption rate is highest in the first 2 min of feeding (Table 2), which may be an important variable in future feeding studies (e.g. effect of genotype, developmental stage, social hierarchy on feeding rate). The image analysis software allows large amounts of feeding data to be collected and analyzed. We found a significant effect of assay day on average consumption rate, perhaps due to the randomization of fish order (e.g. a fish tested last today might be tested first tomorrow, giving it less time to recover an appetite). Nonetheless, we recommend testing over several days to detect clear treatments effects (as seen here). We anticipate this assay could be applied to several small model fish species (e.g. *Medaka* spp.*, Rivulus* spp.*, Fundulus* spp., etc.), with the clear advantage of measuring rate of consumption vs. simply total consumption.

## Supplementary Material

MMC1

MMC2

MMC3

## Figures and Tables

**Table 1:** Mixed effects model ANCOVA for the square root of slopes acquired from regression analysis of individual feeding sessions.

Variable	DF Numerator	DF Denominator	F Value	P Value

Fish Mass	1	19.78	2.1	0.163
Gender	1	19.02	4.21	0.054
Treatment	1	19.37	5.73	**0.027**
Gender* Treatment	1	18.83	0	0.953

**Table 2:** Tukey’s comparison of average minute-to-minute consumption combining genotypes. Minutes that do not share a grouping letter are significantly different. Highest consumption rates are in minutes 1 and 2. There is no interaction between genotype and time (p = 0.137).

MINUTE	N	MEAN	GROUPING

1	22	48.5385	A
2	23	49.2299	A
3	23	34.7619	B
4	23	25.1049	B C
5	23	16.8171	C D
6	23	12.2943	C D
7	23	9.3375	D
8	23	8.8189	D

## Data Availability

Data will be made available on request.

## Appendix A. Supplementary data

Supplementary data to this article can be found online at https://doi.org/10.1016/j.ygcen.2025.114808.

## References

[R1] Brown AJ, Bugescu R, Mayer T, Gata-Garcia A, Kurt G, Woodworth H, Leinninger G, 2017. Loss of action via neurotensin-leptin receptor neurons disrupts leptin and ghrelin-mediated control of energy balance. Endocrinology 158 (5), 1271–1288. 10.1210/en.2017-00122. 28323938 PMC5460836

[R2] Burt JA, Alberto CO, Parsons MS, Hirasawa M, 2011. Local network regulation of orexin neurons in the lateral hypothalamus. Am. J. Physiol.-Regulat. Integrat. Comparat. Physiol. 301 (3), 572–580. 10.1152/ajpregu.00674.2010. 21697524

[R3] de Lecea L, Kilduff TS, Peyron C, Gao XB, Foye PE, Danielson PE, Fukuhara C, Battenberg ELF, Gautvik VT, Bartlett FS, Frankel WN, van den Pol AN, Bloom FE, Gautvik KM, Sutcliffe JG, 1998. The hypocretins: hypothalamus-specific peptides with neuroexcitatory activity. Proc. Natl. Acad. Sci. 95 (1), 322–327. 10.1073/pnas.95.1.322. 9419374 PMC18213

[R4] Elbaz I, Yelin-Bekerman L, Nicenboim J, Vatine G, Appelbaum L, 2012. Genetic ablation of hypocretin neurons alters behavioral state transitions in Zebrafish. J. Neurosci. 32 (37), 12961–12972. 10.1523/JNEUROSCI.1284-12.2012. 22973020 PMC6703801

[R5] Dyachuk V, 2024. The role and mechanisms of the hypocretin system in zebrafish (Danio rerio). Int. J. Mol. Sci. 26 (1), 256. 39796111 10.3390/ijms26010256 PMC11719587

[R6] Furutani N, Hondo M, Kageyama H, Tsujino N, Mieda M, Yanagisawa M, Shioda S, Sakurai T, 2013. Neurotensin co-expressed in orexin-producing neurons in the lateral hypothalamus plays an important role in regulation of sleep/wakefulness states. PLoS One 8 (4). 10.1371/journal.pone.0062391. PMC3631195 23620827

[R7] Gruzdeva O, Borodkina D, Uchasova E, Dyleva Y, Barbarash O, 2019. Leptin Resistance: underlying mechanisms and diagnosis. Dove Medical Press 12 (1), 191–198. 10.2147/dmso.s182406. PMC6354688 30774404

[R8] Jain MR, Horvath TL, Kalra PS, Kalra SP, 2000. Evidence that NPY Y1 receptors are involved in stimulation of feeding by orexins (hypocretins) in sated rats. Regul. Pept. 87 (1–3), 19–24. 10.1016/s0167-0115(99)00102-0. 10710284

[R9] Jordi J, Guggiana-Nilo D, Soucy E, Song EY, Wee CL, Engert F, 2015. A high-throughput assay for quantifying appetite and digestive dynamics. Am. J. Physiol.-Regulatory, Integrat. Comparat. Physiol. 10.1152/ajpregu.00225.2015. PMC4538228 26108871

[R10] Levitas-Djerbi T, Appelbaum L, 2017. Modeling sleep and Neuropsychiatric Disorders in Zebrafish. Curr. Opin. Neurobiol. 44, 89–93. 10.1016/j.conb.2017.02.017. 28414966

[R11] Levitas-Djerbi T, Yelin-Bekerman L, Lerer-Goldshtein T, Appelbaum L, 2015. Hypothalamic leptin-neurotensin-hypocretin neuronal networks in Zebrafish. J. Comp. Neurol 523 (5), 831–848. 25421126 10.1002/cne.23716

[R12] Lewandowski N 2024. (Zebrafish-Hypocretin-Receptor-2-Github https://github.com/Nathanlewandowski/Zebrafish-Hypocretin-Receptor-2/blob/main/Counting%20Brine%20Shrimp-NathanLewandowski-The%20University%20of%20Akron.py).

[R13] Mashhadi Z, Huff Towle H, Schneider C, Davies SS, 2020. A simple and rapid method to measure food intake in fish using brine shrimp. Zebrafish 17 (3), 229–232. 10.1089/zeb.2019.1820. 32125964

[R14] Panula P, 2010. Hypocretin/orexin in fish physiology with emphasis on zebrafish. Acta Physiol. 198 (3), 381–386. 10.1111/j.1748-1716.2009.02038.x. 19723028

[R15] Prober DA, Rihel J, Onah AA, Sung R-J, Schier AF, 2006. Hypocretin/orexin overexpression induces an insomnia-like phenotype in zebrafish. J. Neurosci. 26 (51), 13400–13410. 10.1523/jneurosci.4332-06.2006. 17182791 PMC6675014

[R16] Risner ML, Lemerise E, Vukmanic EV, Moore A, 2006. Behavioral spectral sensitivity of the zebrafish (Danio rerio). Vision Res. 46 (2006), 2625–2635. 16564068 10.1016/j.visres.2005.12.014

[R17] Sakurai T, Amemiya A, Ishii M, Matsuzaki I, Chemelli RM, Tanaka H, Williams SC, Richardson JA, Kozlowski GP, Wilson S, Arch JR, Buckingham RE, Haynes AC, Carr SA, Annan RS, McNulty DE, Liu WS, Terrett JA, Elshourbagy NA, Bergsma DJ, 1998. Orexins and orexin receptors: a family of hypothalamic neuropeptides and G protein-coupled receptors that regulate feeding behavior. Cell 92 (4), 573–585. 10.1016/s0092-8674(00)80949-6. 9491897

[R18] Siegel JM, 2004. Hypocretin (OREXIN): role in normal behavior and neuropathology. Annu. Rev. Psychol. 55 (1), 125–148. 10.1146/annurev.psych.55.090902.141545. 14744212 PMC8765219

[R19] van den Pol AN, Gao X-B, Obrietan K, Kilduff TS, Belousov AB, 1998. Presynaptic and postsynaptic actions and modulation of neuroendocrine neurons by a new hypothalamic peptide Hypocretin/orexin. J. Neurosci. 18 (19), 7962–7971. 10.1523/jneurosci.18-19-07962.1998. 9742163 PMC6793026

[R20] Yokobori E, Kojima K, Azuma M, Kang KS, Maejima S, Uchiyama M, Matsuda K, 2011. Stimulatory effect of intracerebroventricular administration of orexin a on food intake in the zebrafish. Danio Rerio. Peptides 32 (7), 1357–1362. 10.1016/j.peptides.2011.05.010. 21616109

[R21] Zhang X, Zhang Z, Zhao Q, Lou X, 2020. Rapid and efficient live zebrafish embryo genotyping. Zebrafish 17 (1), 56–58. 10.1089/zeb.2019.1796. 31851585

