# Supplementary figures and images for: Genotype for hypocretin receptor (hcrtr2) affects appetite in zebrafish
Source: Gen Comp Endocrinol. Author manuscript; Available in PMC 2026 Jul 6. (PMC13334432; doi:10.1016/j.ygcen.2025.114808)

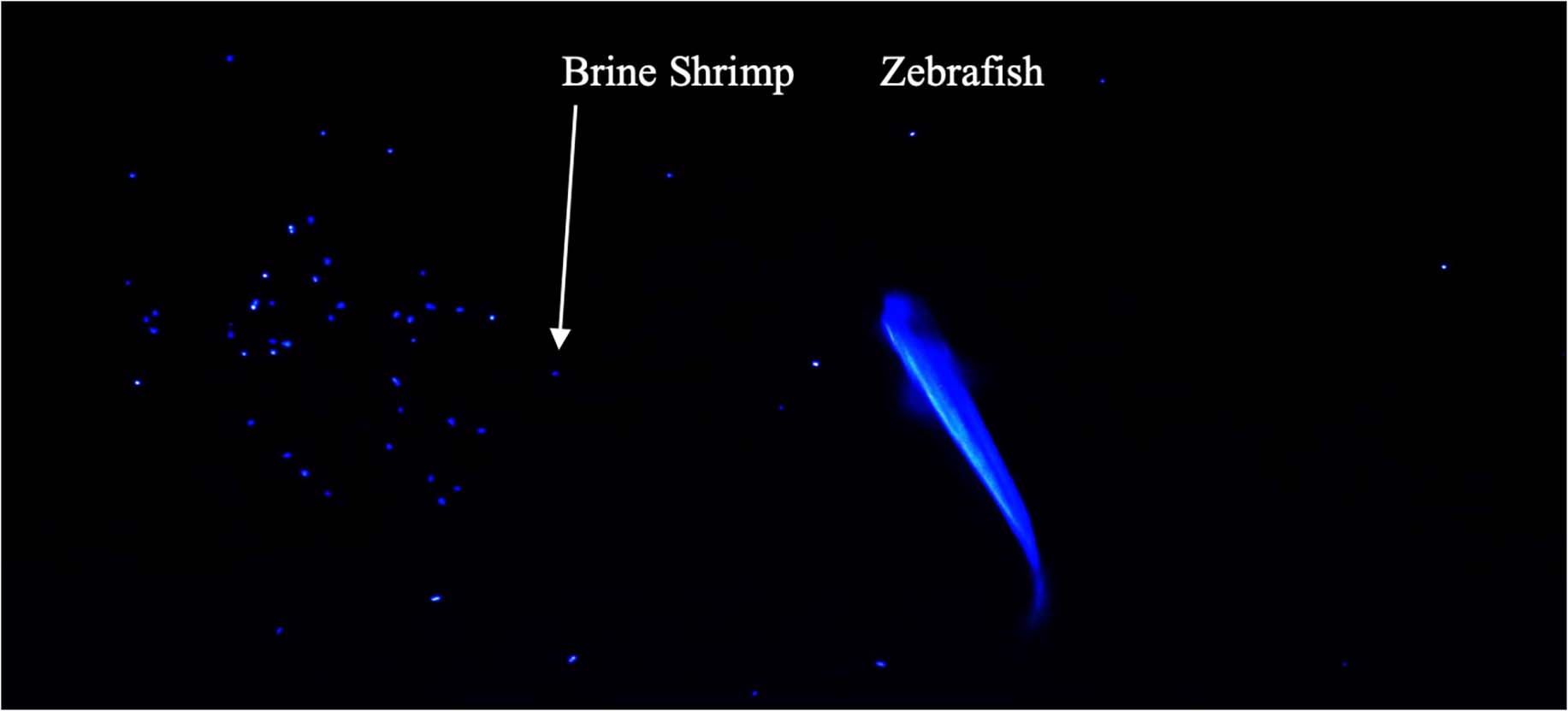

Supplement: MMC1 [file NIHMS2184173-supplement-MMC1.jpg]

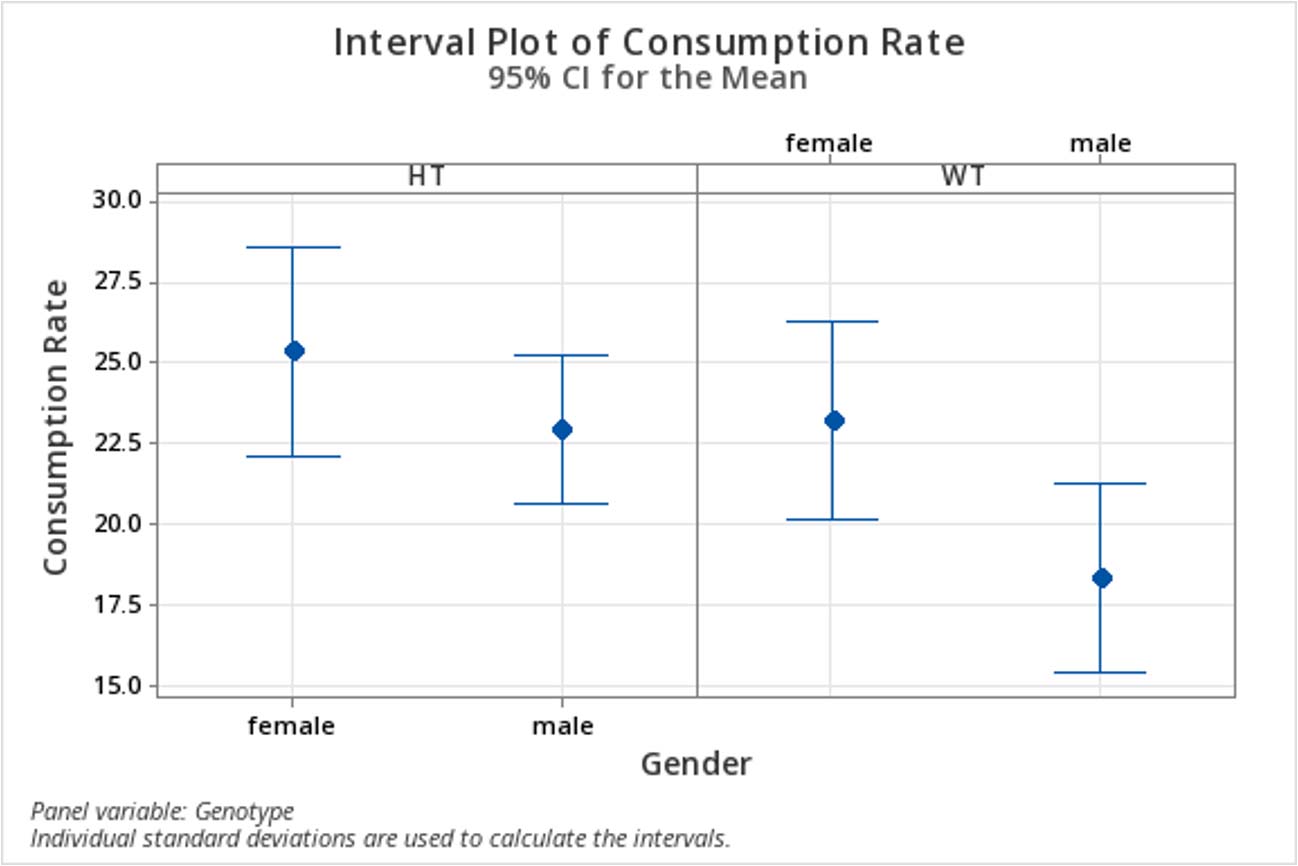

Supplement: MMC2 [file NIHMS2184173-supplement-MMC2.jpg]

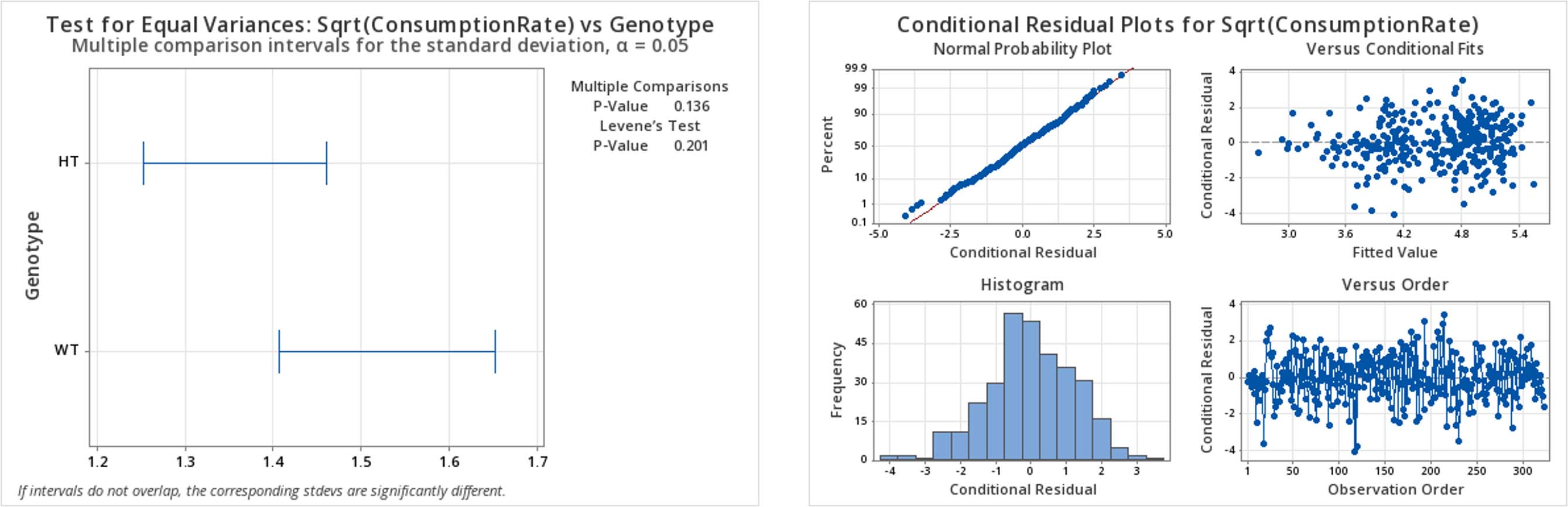

Supplement: MMC3 [file NIHMS2184173-supplement-MMC3.jpg]
